# Fulminant COVID-19-Associated Myocarditis in an Otherwise Healthy Female

**DOI:** 10.7759/cureus.12736

**Published:** 2021-01-16

**Authors:** Gabriela C Milla-Godoy, Robin Park, Weizheng Jiang, Martin W Hartkopf, Thomas Treadwell

**Affiliations:** 1 Internal Medicine, MetroWest Medical Center, Tufts University School of Medicine, Framingham, USA; 2 Infectious Disease, MetroWest Medical Center, Tufts University School of Medicine, Framingham, USA

**Keywords:** fulminant myocarditis, cardiac troponin, coronavirus disease 2019 (covid-19)

## Abstract

An outbreak of a severe respiratory illness caused by a novel coronavirus that began in China in late 2019 has become a pandemic. We report the case of COVID-19-associated myocarditis in a 45-year-old healthy female who presented with solely gastrointestinal symptoms. Initial investigations revealed ST-segment elevations in her electrocardiogram (EKG), elevated troponin levels, and a positive severe acute respiratory syndrome coronavirus 2 (SARS-Cov-2) reverse transcription-polymerase chain reaction (RT-PCR). Subsequently, she had rapid deterioration with the development of cardiogenic shock within hours of admission to a community hospital in Massachusetts. This case highlights an atypical presentation of COVID-19 with a fulminant course in this emerging and evolving disease.

## Introduction

In December 2019, an outbreak of pneumonia caused by severe acute respiratory syndrome coronavirus 2 (SARS-CoV-2) was first identified in a seafood market in Wuhan, China [1]. Subsequently, coronavirus disease 2019 (COVID-19) spread to multiple countries and was declared a pandemic by the World Health Organization in March 2020 [2]. Currently, as of December 30, 2020, there have been 19,432,125 infections in the United States [3] and 352,558 in the state of Massachusetts [4].

The virus mainly causes lung injury. However, multiple other manifestations have been described, including cardiovascular injury and coagulopathy, where the mechanisms have yet to be determined [5]. We present a rare case of an otherwise healthy female patient who presented with gastrointestinal disturbances, ST-segment elevations, and then the development of fulminant myocarditis.

This article was previously presented as a clinical vignette abstract electronic poster presentation for the American College of Physicians, Massachusetts Chapter Meeting on Saturday, October 10, 2020.

## Case presentation

A 45-year-old African female nursing assistant presented to the emergency department with four days of diarrhea, nausea, and vomiting. She endorsed recent contact with COVID-19-positive patients. She denied cough, shortness of breath, chest pain, and fever. She was a non-smoker and was not on any medications. Vital signs showed a blood pressure of 113/85 mm Hg, heart rate of 116 beats per minute (bpm), respiration of 18 breaths per minute, a temperature of 96.0 degrees Fahrenheit, and oxygen saturation of 98% on room air. Her examination was remarkable for an overweight, diaphoretic female in distress with abdominal tenderness and cold and clammy extremities.

**Figure 1:**
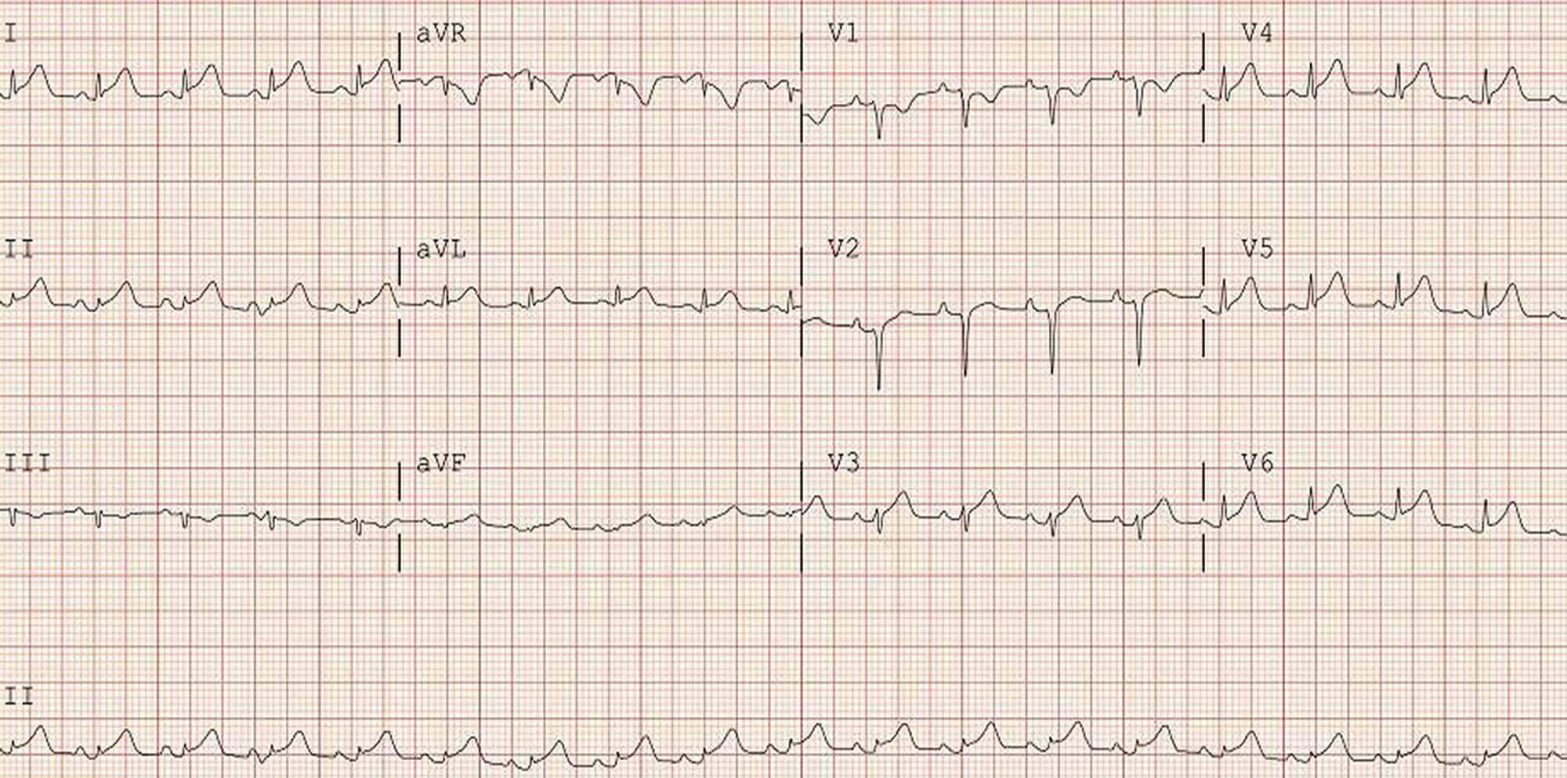
12-lead electrocardiogram (EKG) showing diffuse ST-segment elevations

An electrocardiogram (EKG) showed sinus tachycardia and diffuse ST-segment elevation in I, II, augmented Vector Left (aVL), and V3-V6 (Figure 1).

**Figure 2:**
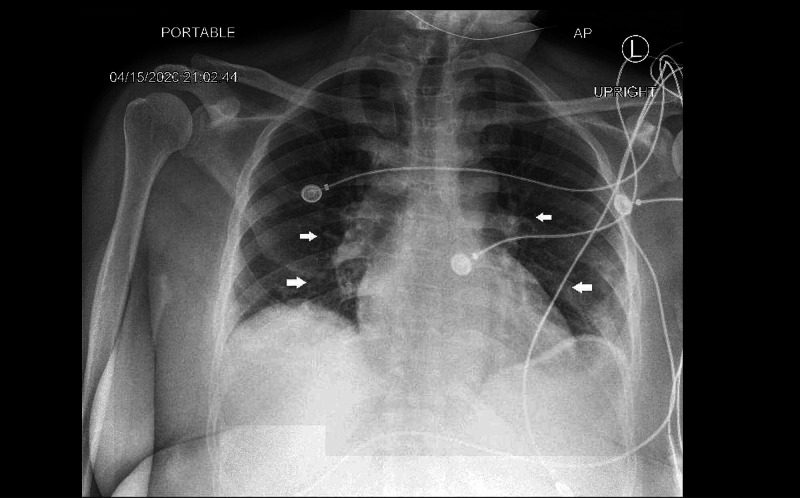
Chest x-ray showing patchy bilateral infiltrates (arrows)

Laboratory testing was remarkable for a white blood cell count of 3.8 10^3^/uL (reference range: 4.0 - 11.0 10^3^/uL), platelets 144 10^3^/uL, (reference range 150 - 400 10^3^/uL), international normalized ratio (INR) 1.26 (reference range 0.8 - 1.1), lactic acid 2.97 mmol/L (reference range: 0.5 - 2.00 mmol/L), ferritin 562 ng/mL (reference range: 13 - 150 ng/mL), C-reactive protein (CRP) 24.1 mg/L, (reference range: 0 - 5 mg/L); serum aspartate aminotransferase (AST) 141 U/L (reference range: 10 - 34 U/L), lactate dehydrogenase (LDH) 464 U/L (reference range: 135 - 235 U/L), creatine kinase (CK) 693 U/L (reference range: 26 - 192 U/L), N-terminal pro-B-type natriuretic peptide (NT-proBNP) 4,585 pg/mL (reference range: 0 - 450 pg/mL), and troponin T 0.43 ng/mL (reference: 0 - 0.02). The chest x-ray showed patchy infiltrates bilaterally (Figure 2). SARS-CoV-2 reverse transcription-polymerase chain reaction (RT-PCR) was positive.

The patient initially received 2 liters of normal saline, hydroxychloroquine 400 mg, aspirin 325 mg, and atorvastatin 80 mg for presumptive COVID-19-associated myocarditis. She improved symptomatically and remained hemodynamically stable. Two hours later, she became lethargic with a heart rate of 130 bpm. Pulses were unmeasurable. She received another liter bolus of normal saline and was immediately transferred to the intensive care unit. A bedside echocardiogram showed a left ventricular ejection fraction of approximately 10% with global hypokinesis and no pericardial effusion.

Ultimately, the patient developed cardiogenic shock. She was intubated and started initially on norepinephrine and subsequently required additional pressor support with phenylephrine and dopamine. She received intravenous immunoglobulin and methylprednisolone. She subsequently became asystolic. She received four doses of intravenous (IV) epinephrine and 1 ampule of IV bicarbonate. The patient expired after 15 minutes of resuscitation.

## Discussion

SARS-Cov-2 has caused significant morbidity and mortality worldwide. It is broadly known that the main target organ is the lung. However, recent case reports have reported cardiovascular involvement, including fulminant myocarditis [6-7]

Fulminant myocarditis is often preceded by a viral prodrome and characterized by sudden and severe diffuse cardiac inflammation. It is usually fatal, resulting from cardiogenic shock, arrhythmias, or multiorgan failure [8]. The proposed mechanisms of myocardial injury due to COVID-19 rely on the angiotensin-converting enzyme 2 (ACE2) receptor as its point of entry. Its inhibition may prevent the breaking and accumulation of angiotensin II, a proinflammatory factor in the lung, resulting in systemic inflammation, cytokine release, and procoagulant activity [9].

Fulminant myocarditis is a challenging diagnosis as its clinical presentation may overlap with pericarditis and acute coronary syndrome. Troponin elevation is a common finding. Although an elevated troponin level in a COVID-19 infection does not necessarily mean fulminant myocarditis, its high clinical significance relies on the fact that it has been shown that elevation of this marker is associated with higher mortality, even in those without underlying cardiovascular disease [10-11]. The most common EKG findings include ST-segment elevations and PR segment depression. Echocardiography findings may range from focal wall motion abnormalities to global hypokinesis with systolic dysfunction [12]. Currently, there are no established therapeutic agents for COVID-19 myocarditis, although some cases have reported therapeutic benefits associated with corticosteroids and immunoglobulin G [13-14].

This case, and many others with reported SARS-CoV-2 myocarditis, give insight into the importance of close monitoring in patients that present with elevated cardiac biomarkers as this may be an indicator for a poor prognosis.

## Conclusions

Fulminant myocarditis may be a complication associated with COVID-19 infection. A mechanism of cardiac injury by SARS-CoV-2 has yet to be determined. One that has been proposed may be attributed to its inhibition of the ACE2 receptor as its point of entry, resulting in systemic inflammation and cytokine release. Elevated troponin T in COVID-19 infection may be a marker of higher mortality and poor prognosis. Regardless of the clinical presentation, close monitoring is paramount for the early recognition of severe complications. Further large-scale studies are needed in order to determine the role of intravenous immunoglobulin G (IVIG) and corticosteroid therapy on patients with COVID-19-associated myocarditis. 
